# Effects of Different pH Levels on the Structural and Functional Properties of Proteins of *Phaeodactylum tricornutum*

**DOI:** 10.3390/molecules29133139

**Published:** 2024-07-01

**Authors:** Yanli Wang, Laijing Zhu, Zhunyao Zhu, Meng Liu, Xiangzhong Zhao

**Affiliations:** School of Food Science and Engineering, Qilu University of Technology (Shandong Academy of Sciences), Jinan 250353, China; 15106614358@163.com (Y.W.); 15725152458@163.com (L.Z.); 18366260356@163.com (Z.Z.); 15864530661@163.com (M.L.)

**Keywords:** *Phaeodactylum tricornutum* protein, different pH levels, structure, functional properties

## Abstract

*Phaeodactylum tricornutum* is identified by its capacity for rapid growth, reproduction, and in vitro cultivation, as well as the presence of a range of high-value active compounds, including proteins, with potential food applications. The objective of this study was to investigate the effects of pH shift treatments (pH of 3, 5, 7, 9, and 11) on the structural and functional properties of the *Phaeodactylum tricornutum* protein (PTP). The molecular weight of the PTP was predominantly distributed within the following ranges: below 5 kDa, 5–100 kDa, and above 100 kDa. Compared to the acidic environment, the PTP demonstrated higher solubility and greater free sulfhydryl group content in the alkaline environment. Additionally, PTP had a smaller particle size and higher thermal stability in alkaline environments. The PTP exhibited superior foaming ability (135%), emulsification activity index (3.72 m^2^/g), and emulsion stability index (137.71 min) in alkaline environments. The results of this investigation provide a foundation for the future development and application of the PTP in the food industry.

## 1. Introduction

The requirement to find new and alternative sources of protein has been increasing in recent years. With global warming, declining biodiversity, diminishing water resources, and increasingly discerning tastes in food due to a growing worldwide population, the search for sustainable sources of protein has become imperative [1]. Microalgae may be considered an innovative and promising food ingredient, offering a high level of nutrients. In addition to high-value proteins, long-chain polyunsaturated fatty acids, and vitamins, microalgae contain carotenoids, minerals, and other bioactive compounds [2]. Microalgae are a class of extremely vigorous species that can live in a variety of environments such as deserts, permafrost, and volcanic water [3] and are characterized by strong carbon sequestration, fast growth rates, diverse cultivation methods, and short growth cycles; they can also be cultured industrially. Microalgae represent a particularly promising source of new food products when considered alongside the relatively low land use area required compared to traditional animal husbandry practices, as well as when considered alongside the lack of herbicides and pesticides required compared to traditional crops, the lack of land conditions required compared to traditional crops, and the lack of seasonality limitations compared to traditional crops. Microalgae are regarded as a highly promising source of bioactive compounds in food, exhibiting a high degree of diversity in their chemical composition. They are particularly rich in a wide range of bioactive substances, including proteins, polyunsaturated fatty acids, pigments, polysaccharides, vitamins, polyphenols, and phytosterols [4].

*Phaeodactylum tricornutum* is a unicellular alga with ovate, fusiform, and tridentate types. It is found exclusively in marine environments [5]. Despite its small cell size, *Phaeodactylum tricornutum* cells are rich in protein and other nutrients, making them easily digestible and absorbable by animal larvae. At the same time, *Phaeodactylum tricornutum* exhibits rapid growth, adaptability to temperature, salinity, light intensity, acidity, and alkalinity, and high-density cultivation potential, which collectively render it an exceptionally high-quality protein bait and a prevalent algal bait utilized in aquaculture. *Phaeodactylum tricornutum* is rich in polyunsaturated fatty acids, fucoxanthin, and other active substances, and it contains ten times more fucoxanthin than other macroalgae, with anti-inflammatory, antioxidant, anti-obesity, and anticancer activities [6]. *Phaeodactylum tricornutum* is of great interest due to its short reproduction cycle and high accumulated lipid content, being especially rich in omega-3 polyunsaturated fatty acids (omega-3 PUFAs), including EPA and DHA. It is well established that ω-3 PUFAs are beneficial to human health, preventing and treating many chronic diseases such as inflammation, diabetes, and cardiovascular diseases [7]. *Phaeodactylum tricornutum* contains 34.8–39.6% crude protein, and the extracts from *Phaeodactylum tricornutum* have good emulsification and rheological properties [8].

pH adjustment is an alteration technique that modifies the structural composition and improves the functional attributes of proteins [9]. This process leads to modifications in the electronic configuration and spatial conformation of the protein, allowing it to unfold and refold at the appropriate pH value [10]. The adjustment of protein pH has been found to enhance the emulsifying, gelating, and foaming properties of proteins and alter the solubility of animal and plant proteins [11]. The structural features, functions, and physicochemical properties of proteins are affected by changes in pH. The effect of pH on the structural features, functions, and physicochemical properties of the PTP is less well-documented. The objective of the present study was to ascertain the impact of different pH levels on the structural and functional properties of isolated proteins derived from *Phaeodactylum tricornutum.*

## 2. Results and Discussion

### 2.1. Amino Acid Composition of the PTP

The amino acid composition of proteins is very important in nutritional and functional terms [12]. The amino acid composition of the PTP is shown in Table 1. The total content of essential amino acids in the PTP (45.44 g/100 g) was found to be higher than that of essential amino acid soy protein (39.62 g/100 g). Meanwhile, the content of several essential amino acids (Thr, Val, Leu, Lys, and His) in the PTP was higher than the levels of essential amino acids recommended for adults by the FAO/WHO [13]. Only lysine (4.87 ± 0.11 g/100 g) differed slightly from FAO/WHO recommendations for children. Aspartic acid (Asp) and glutamic acid (Glu) have been shown to promote antioxidant function by providing additional electrons during free radical interactions [14]. The levels of aspartic acid (Asp; 8.09 g/100 g) and glutamic acid (Glu; 8.26 ± 0.16 g/100 g) were higher in the PTP than the other amino acids, in agreement with the results of a previous study [15]. Meanwhile, according to Phat [16], high levels of glutamic acid and aspartic acid produce specific flavors and umami tastes, suggesting that the PTP has the potential to enhance the umami taste of foods. In addition, the PTP also had a high proportion of hydrophobic amino acids (39.25 g/100 g), an indication that the PTP has the potential to form a spherical structure [17].

### 2.2. Molecular Weight (MW)t Analysis of the PTP

**Figure 1:**
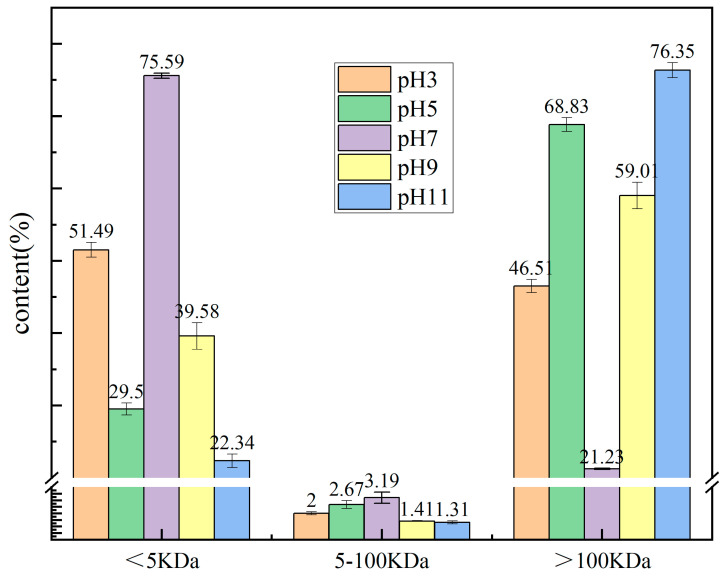
Molecular weight distribution of the PTP at pH 3, 5, 7, 9, and 11.

As illustrated in Figure 1, the majority of the predominant MW at a pH of 3 was below 5 kDa (51.49% ± 1.02) and above 100 kDa (46.51% ± 0.92). Conversely, the molecular weight at a pH of 5 exhibited a lower proportion of molecules below 5 kDa and a higher proportion above 100 kDa than at a pH of 3. The disruption of electrostatic interactions between protein molecules at a pH of 5 may be the causal factor in the subsequent aggregation of these molecules into protein aggregates [18]. In the context of alkaline conditions, the molecular weight exhibits a decrease with increasing pH below 5 kDa and an increase above 100 kDa. This indicates that changing the pH has a significant effect on the molecular weight of the PTP. In line with the observations of Lutz et al. [15], the molecular weights of the PTPs with varying pH values exhibited a distribution in the following ranges: below 5 kDa, between 5 and 100 kDa, and above 100 kDa (Figure 1). 

### 2.3. Solubility

**Figure 2:**
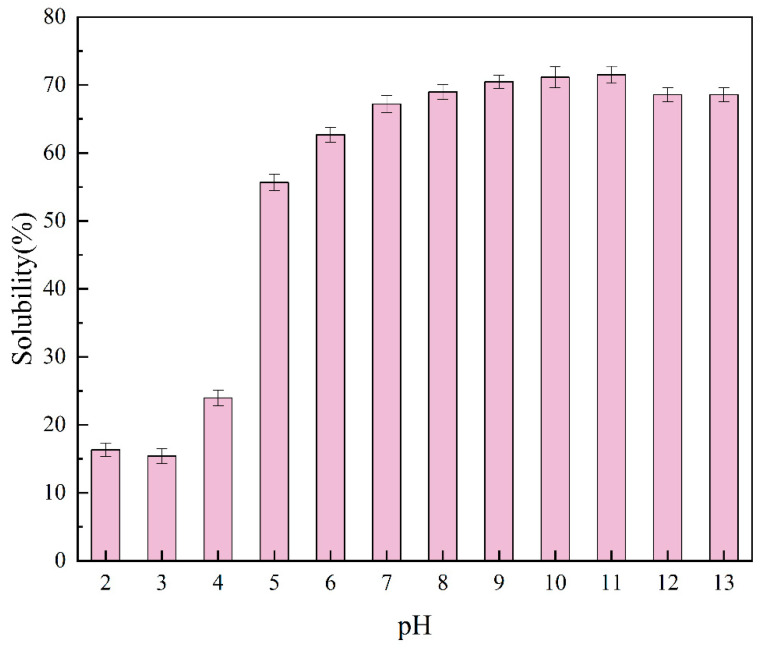
Solubility of the PTP at pH values of 2–13.

The solubility of proteins represents an important indicator of protein denaturation and aggregation, which in turn affects their functional properties [19]. As expected, pH had a significant effect on the solubility of proteins. At pH levels of 2–13, the solubility of the PTP showed an appreciable rise from 15.41% to 71.49%. At a pH of 3 (Figure 2), this increase reached a minimum (15.41%). This is consistent with the findings obtained by Chen [20], among others, showing that most proteins have minimum solubility values near isoelectric points. Similarly, spirulina proteins were found to be more soluble in alkaline (98.07%) than acidic (3.23%) environments [21].

The net charge on the protein molecules accounts for the low solubility of the PTP at pH levels of 2.0–3.0, which is near the isoelectric point. Aggregation and precipitation of proteins are a result of this process. This is caused by a decrease in the electrostatic repulsion between the protein molecules [22]. However, protein unfolding is favorably facilitated by alkaline circumstances (pH >7.0), which also increase the electrostatic repulsion between protein molecules by raising their net charges, thus improving protein–water interactions and protein solubility. The high solubility of the PTP in alkaline environments showed that the PTP could be appropriate for use in alkaline formulation foods. 

### 2.4. Free Sulfhydryl Groups

**Figure 3:**
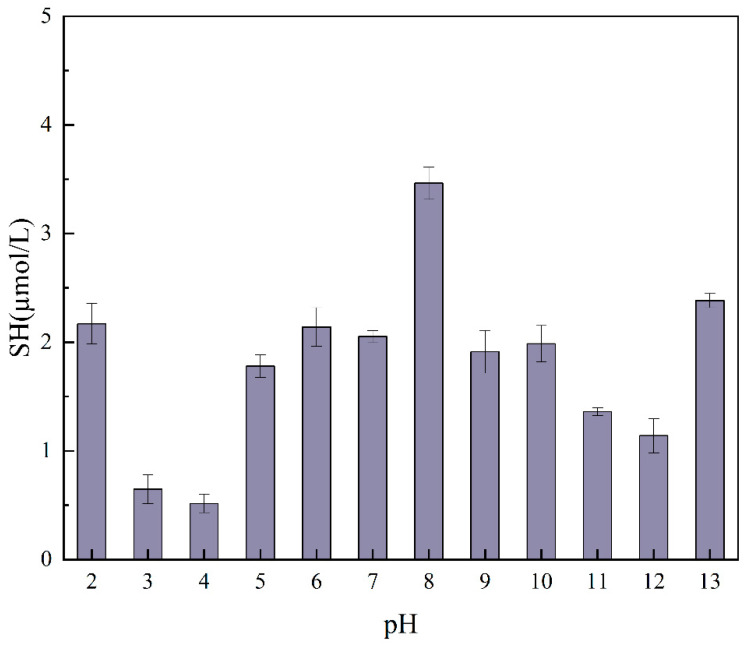
Free sulfhydryl content of the PTP at pH levels of 2–13.

Figure 3 illustrates the SH of the PTP treated at varying pH values. It can be observed that the pH conversion treatment resulted in the protein undergoing partial unfolding, thereby exposing the sulfhydryl groups within the protein. Conversely, the pH conversion treatment resulted in the disruption of the S-S bonds within the protein, causing severe damage to the structure of the protein [23]. The content of free sulfhydryl groups was higher at a pH of 8, whereas the content of free sulfhydryl groups decreased at pH levels of 4 and 3. The reason for this phenomenon may be that near the protein isoelectric point, there is relatively little electrostatic repulsion between proteins and a tendency for protein particles to aggregate, preventing the exposure of sulfhydryl groups [23]. This also leads to an increase in particle size. Previous studies have shown that changes in the free SH content of proteins are associated with conformational changes and deformation of the protein. In addition, higher levels of SH in proteins have been shown to promote the formation and stabilization of foam films, which in turn increases the stability of the foam [24]. The results are consistent with those obtained in studies on foam formation and foam stability.

### 2.5. Secondary Structure

**Figure 4:**
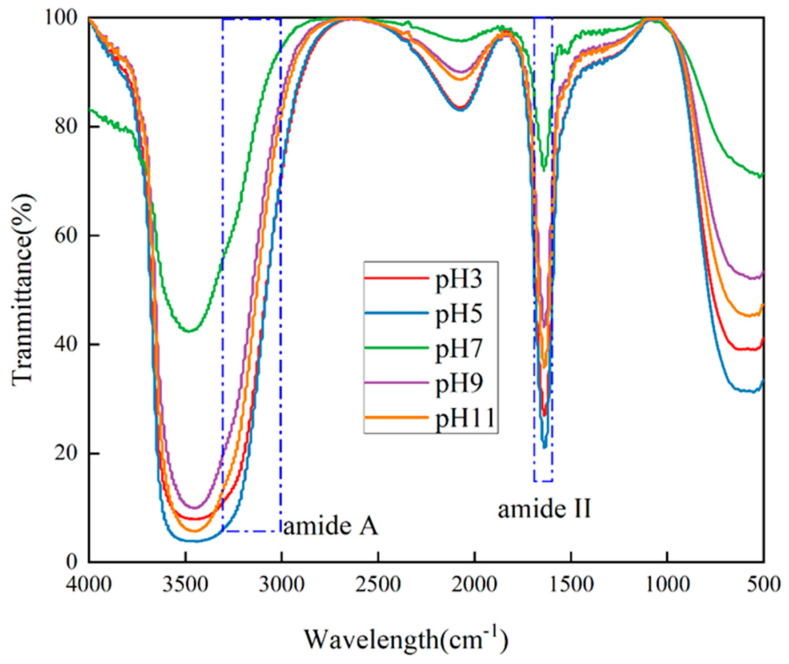
Fourier transform infrared (FTIR) spectra of the PTP at pH 3, 5, 7, 9, and 11.

FTIR spectroscopy is a technique that is used to analyze the chemical structure of samples. It involves observing the absorption of infrared radiation in terms of wavelength and intensity. The non-destructive and convenient nature of this approach allows for minimal sample preparations and can be used in a wide range of situations. As an example, FTIR has been used extensively in the study of the secondary structure of peptides and proteins, such as in [20]. The data from the FTIR analysis for the different pH treatments are shown in Figure 4. The amide I region between 1600 cm^−1^ and 1700 cm^−1^ can be used to determine the secondary structure of proteins, including α-helices (1330–1295 cm^−1^), β-turns (1295–1270 cm^−1^), β-folds (1250–1220 cm^−1^), and random coil (1270–1250 cm^−1^), based on molecular vibrations. The secondary structure of proteins can be determined by the analysis of the molecular vibrations that give rise to the following peaks: β-turns, β-folds, and random coil. This is directly related to the conformation of the polypeptide chain and is mainly associated with the C==O stretching vibration. Amide I (1700–1600 cm^−1^) is most sensitive to the secondary structure of the protein. However, it is susceptible to strong interference from the water vibrational band. Amide A, associated with the -NH stretching vibration coupled with the intramolecular hydrogen bond, is responsible for the absorption peaks observed at 3000–3500 cm^−1^. The bond is related to the -NH stretching vibration coupled with the intramolecular hydrogen bond [25]. The peak intensities of the PTP at both amide I and amide A showed a significant increase after pH treatment, indicating that pH treatment had a profound effect on the strength of the intramolecular hydrogen bonding and the interaction patterns of the amide moieties, leading to the appearance of specific spectral changes.

**Figure 5:**
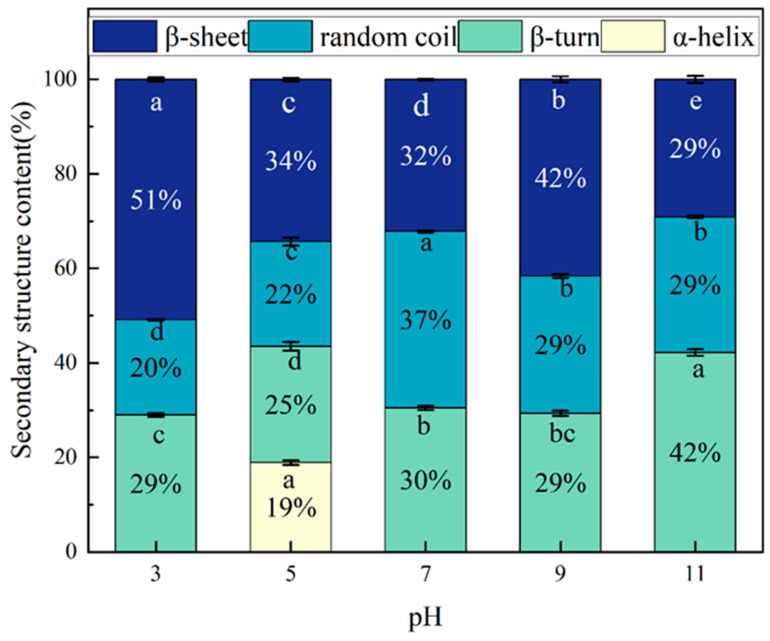
Secondary structure composition of the PTP at pH 3, 5, 7, 9, and 11. Different letters indicate significant differences between groups (*p* < 0.05).

The secondary structure of the PTP at different pH values is shown in Figure 5, which shows that the secondary structure content shows an irregular variation with pH. The β-folding at a pH of 3 was found to be significantly higher than in the other samples. Previous studies have demonstrated that proteins exhibiting a high proportion of β-folding tend to possess rigid structures and exhibit low solubility [26], consistent with the findings observed with a lower solubility at a pH of 3. In contrast, the content of the random coil was significantly diminished in the other samples relative to a pH of 7, indicative of a pH shift treatment-induced transformation of the secondary structure of the PTP from a disordered state to an ordered state.

### 2.6. Intrinsic Fluorescence Spectroscopy

**Figure 6:**
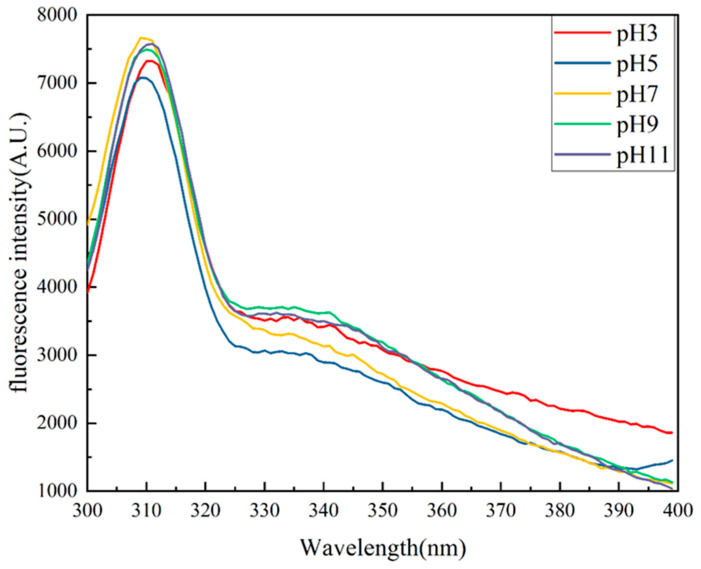
Intrinsic fluorescence spectra of the PTP at pH 3, 5, 7, 9, and 11.

Intrinsic fluorescence spectroscopy (Figure 6) was used to investigate the tertiary structure of the *Phaeodactylum tricornutum* protein. Intrinsic fluorescence spectra are a commonly used method to characterize changes in the tertiary structure of proteins [27], are a consequence of the excitation of aromatic amino acid residues (particularly tryptophan), and depend on the polarity of the environment surrounding the aromatic amino acid residues. At a pH of 7, alkali-treated samples showed a red shift in the maximum emission, while fluorescence intensity declined as pH increased. This is thought to be the result of changes in protein structure and the exposure of tryptophan residues to a polar milieu. The results demonstrated that the acidic treatment exhibited a lower fluorescence intensity compared to the alkaline treatment. Additionally, a blue shift in the maximum emission wavelength of the samples was observed. These results could be explained by the proximity of the isoelectric point of the PTP (pH of approximately 3.5) and the presence of a large amount of insoluble precipitates. 

### 2.7. Surface Hydrophobicity

**Figure 7:**
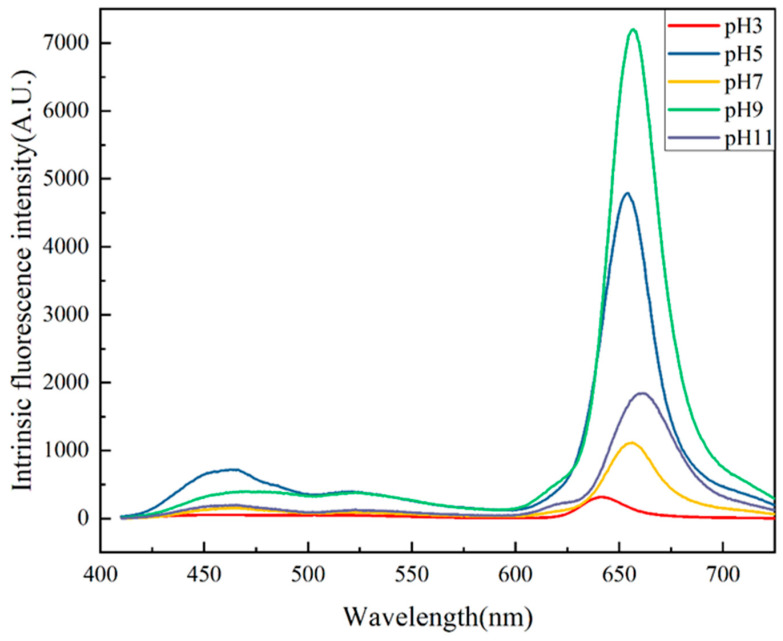
Surface hydrophobicity of the PTP at pH 3, 5, 7, 9, and 11.

Since proteins have a high affinity for hydrophobic surfaces, ANS can be used to assess changes in the hydrophobic sites and, consequently, the structure of the proteins that are exposed. Peaks in the fluorescence spectra of proteins can be used as a reflection of the intensity of hydrophobicity and can further reveal the spatial structure and functional properties of the protein [28]. As shown in Figure 7, the surface hydrophobicity of the PTP showed a significant increase under alkaline treatment. Regardless of the expanded intramolecular electrostatic repulsion and protein unfolding caused by the alkaline pH, hydrophobic amino acid residues were exposed. Additionally, due to a greater shift in surface hydrophobicity in the alkaline treatment compared to the acidic treatment, the alkaline pH treatment caused more significant conformational changes in the PTP. At a pH of 3.0, the exposure of hydrophobic groups led to embedding hydrophilic groups, resulting in decreased solubility of the PTP. A negative correlation between surface hydrophobicity and protein solubility was observed when the protein was away from its isoelectric point. At a pH of 3.0, minimum surface hydrophobicity was observed as the protein particles precipitated and aggregated in the vicinity of the isoelectric point, burying the hydrophobic residues. 

### 2.8. Thermal Properties

DSC is an efficacious instrument for the analysis of the impact of heat on the physical or chemical properties of a substance. In DSC measurements, the temperature at which the maximum peak occurs is typically utilized to ascertain the denaturation temperature, which can be employed as an indicator of the thermal stability of the compound. As illustrated in Table 2, the initial temperatures of denaturation (T_O_) of all the samples were close to 40°C. The ΔH of all samples was greater than that at a pH of 7, which is an indication of the formation of more stable complexes. It can be seen that the degree of order within the structure and the thermal stability of the protein increases as the enthalpy increases, as does the force required to maintain the protein structure [29]. The ΔH of the PTP under acidic conditions was found to be significantly lower than that of the PTP under alkaline conditions, indicating that the PTP is more thermally stable under alkaline conditions. 

### 2.9. Particle Size and Zeta Potential

As illustrated in Table 3, the particle size of the PTP diminishes with an increase in pH. The average particle size of the PTP is at its maximum at a pH of 3 and minimum at a pH of 11, with values of 3199.84 ± 0.47 nm and 1112.11 ± 0.57 nm, respectively. The delta-brown fingerling proteins subjected to a pH change (pH of 11) exhibited a decrease in particle size. This phenomenon can be attributed to the larger contact space between the protein and water molecules, which results in a stronger interaction between them. It is generally accepted that protein aggregates with smaller particle sizes exhibit greater specific surface areas, which in turn enhance their interaction with aqueous solutes. This increases the solubility of the PTP. Furthermore, the reduction in protein particle size facilitates protein adsorption at the oil–water interface, thereby improving its emulsification properties [30].

Zeta potential is a measure of the surface electrical properties of particles in solution. Stronger intermolecular repulsion, lower aggregation, and better stability are observed in systems with higher absolute zeta potential. The absolute zeta potential of the PTP after a pH shift showed an increase with pH, with the highest absolute zeta potential value of 45.33 ± 0.54 mV observed at a pH of 11 due to the change in the tertiary structure of the protein, resulting in a change in the surface charge potential [31].

### 2.10. Foaming Capacity and Foaming Stability

**Figure 8:**
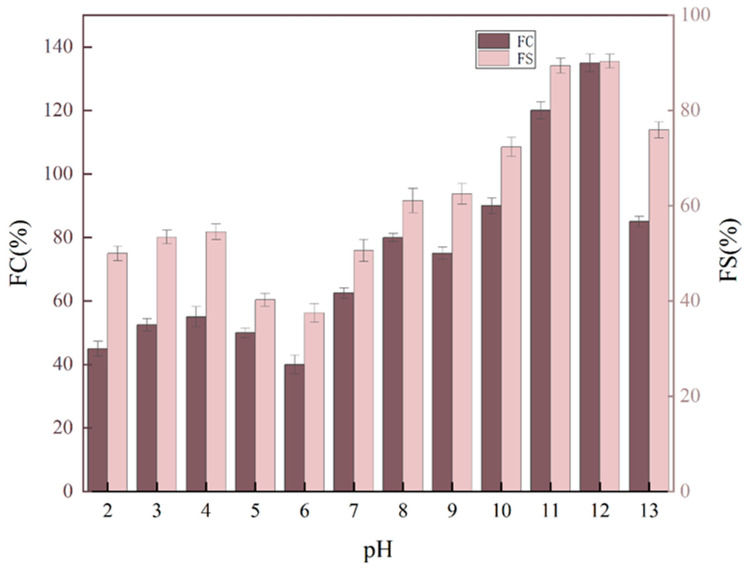
Foamability and foam stability of the PTP at pH levels of 2–13.

The foaming ability of protein isolates is dependent on several factors, including solubility, conformational chain flexibility, and degree of hydrophobicity. Conversely, foaming stability is influenced by rheological behavior, protein–protein interactions, pH, and temperature. Furthermore, the unfolding of proteins facilitates their ability to diffuse at the air–water interface, enabling them to encapsulate air, thereby enhancing their foaming capacity. Figure 8 illustrates the impact of pH change on PTP foaming and foam stability. It demonstrates that foaming capacity was reduced under acidic conditions, which may be attributed to the low solubility of the proteins under acidic conditions. The highest FC was observed at a pH of 12, which was attributed to the alkaline conditions of the protein adsorption rate at the air/water interface, which is faster. The combination of suitable surface hydrophobicity, a smaller particle size, and a larger solubility resulted in the fastest adsorption rate of the PTP at a pH of 12, which in turn led to an improvement in foaming.

### 2.11. Emulsifying Activity and Emulsion Stability

**Figure 9:**
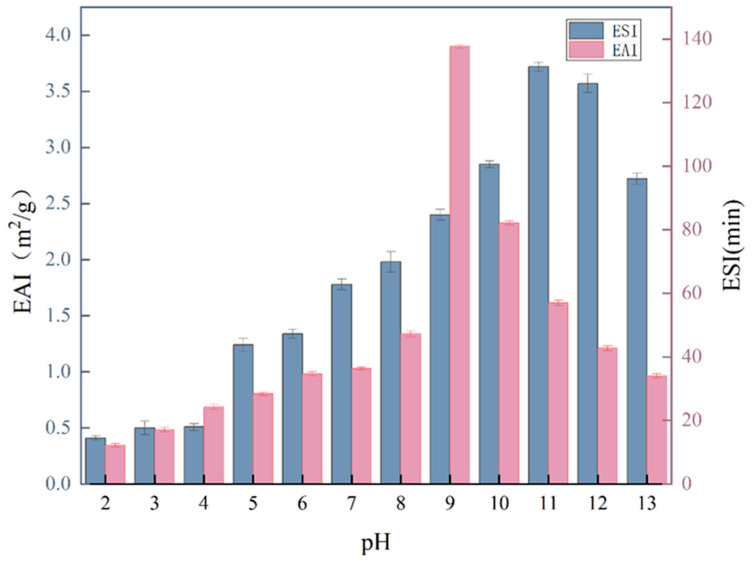
Emulsifiability and emulsion stability of the PTP at pH levels of 2–13.

The emulsification properties of a protein are typically evaluated using two different methods: EAI and ESI. EAI measures the ability of proteins to adsorb at interfaces, while ESI evaluates the properties of the adsorbed layer formed. As illustrated in Figure 9, the emulsification of the PTP is better in alkaline than in acidic environments. It is known that two significant factors affecting the capacity of proteins to emulsify are their solubility and surface hydrophobicity. The performance of protein emulsification is generally more successful at higher values of both of those parameters. The EAI and ESI values of the PTP were the lowest at a pH of 3. Given that the surface charge of the PTP is close to 0 when pH is close to the isoelectric point, flocculation between protein molecules occurs due to electrostatic adsorption, resulting in a decrease in the emulsification stability of the system. 

### 2.12. Antioxidant Activity

#### 2.12.1. ABTS Free Radical Scavenging Activity

**Figure 10:**
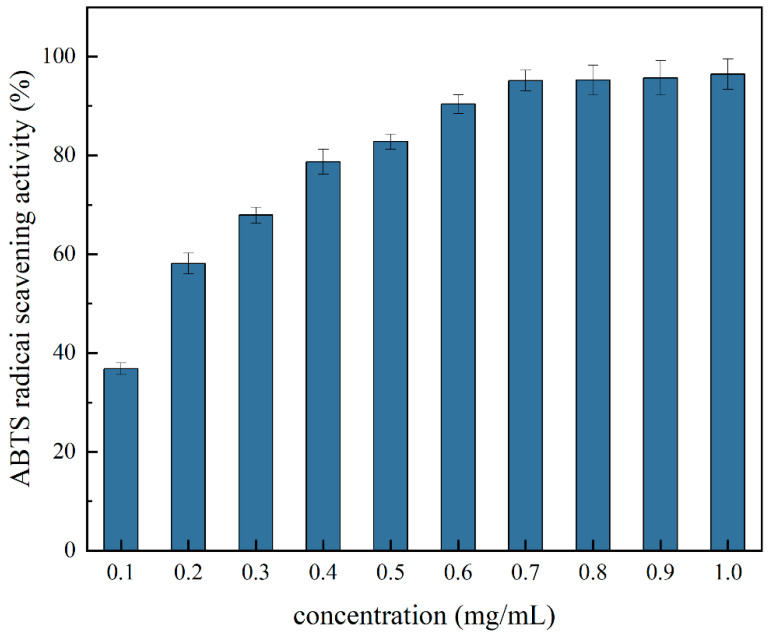
ABTS radical scavenging activity at different concentrations of the PTP.

The ABTS radical scavenging assay, a method based on the transfer of hydrogen atoms and electrons between the ABTS+ radical and the test substance, is the cornerstone for assessing the antioxidant capacity of lipophilic and hydrophilic peptides. As illustrated in Figure 10, the ABTS radical scavenging activity increased with increasing concentration of the PTP. This indicates that the ABTS radical scavenging activity of the PTP is affected by the concentration of the PTP. 

#### 2.12.2. DPPH Free Radical Scavenging Activity

**Figure 11:**
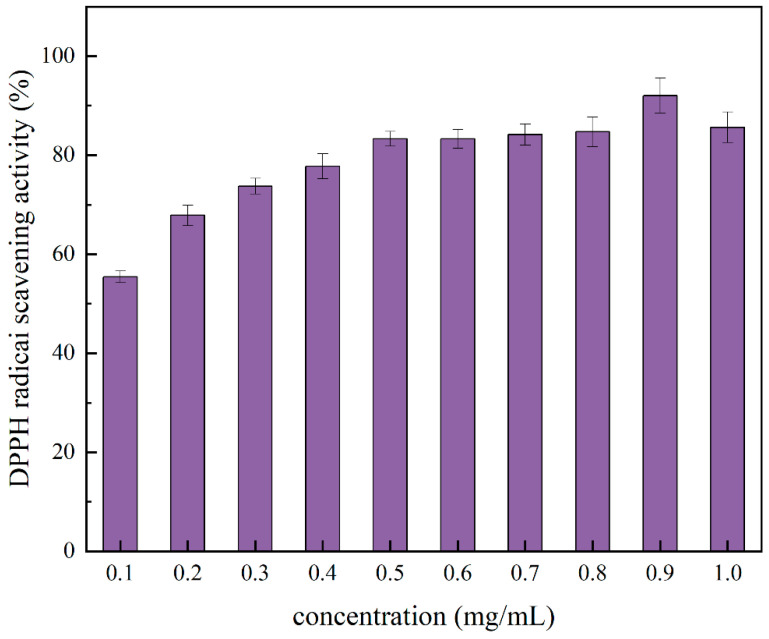
DPPH radical scavenging activity at different concentrations of the PTP.

Figure 11 illustrates the DPPH radical scavenging capacity of the PTP, and the results show that its ability to scavenge DPPH free radicals rises as the concentration of the PTP increases. The PTP exhibited a stronger DPPH radical scavenging capacity at 0.9 mg/mL (92.03 ± 2.52%) and a lower DPPH radical scavenging capacity at 0.1 mg/mL (55.42 ± 1.13%). The results showed that the DPPH radical scavenging activity of the PTP is affected by the concentration of the PTP. 

## 3. Materials and Methods

### 3.1. Materials 

The seaweed powder was procured from Xunshan Group Limited (Weihai, China), while the soybean oil was sourced from a local supermarket in Jinan, Shandong, China. The following reagents were purchased from Sigma-Aldrich Co., Ltd. (Shanghai, China): EDTA, Coomassie Brilliant Blue (R-250), 1-aniline-8-naphthalenesulfonic acid (ANS), and dithiobisnitrobenzoic acid. The reagents potassium bromide, sodium hydroxide, and hydrochloric acid were obtained from Sinopharm Group Co Ltd. (Shanghai, China). Acetonitrile and trichloroacetic acid were purchased from Xilong Science Co. Ltd. (Guangdong, China), and potassium persulphate was purchased from Shanghai McLean Biochemical Technology Co. (Shanghai, China).

### 3.2. Preparation of Phaeodactylum Tricornutum Protein (the PTP)

The preparation of the PTP was carried out according to Aline et al. [32]. The pH of the algae powder suspension (1:45, g/mL) was adjusted to 12.5 with NaOH at a concentration of 1 M, and the suspension was then centrifuged (10,000 r, 20 min) at 55 °C to remove the precipitate. The pH of the supernatant was adjusted to 3.5 (isoelectric point) with 1 mol/L hydrochloric acid and centrifuged again (10,000 r, 20 min) to obtain the precipitate. The obtained precipitate was redispersed in deionized water; then, the dispersion pH was adjusted to 7.0 with 1 M NaOH and lyophilized using a freeze-drier (Scientz-18 N, Ningbo Xinzhi Biotechnology Co., Ltd., Ningbo, China).

### 3.3. Amino Acid Analysis

Amino acids were quantified utilizing a Bio30+ fully automated amino acid analyzer (Biochrom 30+, Biochrom, UK). For this purpose, 40 mg of the PTP lyophilized powder was dispersed homogeneously in 4 mL of 6 mol/L hydrochloric acid. The solution was subjected to hydrolysis at 110 °C under nitrogen for a period of 24 h. Subsequently, the hydrolyzed sample was diluted to 100 mL, with 2 mL being deacidified at 60 °C. The sample was then diluted to 0.22 microns. Amino acid analysis was conducted by inhalation of 0.02 µL at a time.

### 3.4. Molecular Weight Distribution (MW) of the PTP

The method described by Yixuan et al. [33] used to analyze the molecular weight (MW) distribution, with slight modifications. To evaluate the molecular weight distribution, a high-performance liquid chromatography (HPLC) system (Shimadzu, Kyoto, Japan) outfitted with an ultraviolet (UV) detector and a TSK gel filtration column G2000SWXL (Tosoh Bioscience, Yamaguchi, Japan) was used. The lyophilized PTP samples subjected to different pH treatments (pH of 3.0,5.0,7.0,9.0, and 11.0) were dispersed in the mobile phase to obtain a solution with a concentration of 0.2 mg/mL. This solution was then filtered through a filter head with a pore size of 0.22 µm. The mobile phase was injected at a rate of 1 mL per minute at 25 °C with a sample volume of 10.0 µL. It was composed of acetonitrile, water, and trifluoroacetic acid (45:55:0.1, mL: mL: mL) with a volume ratio of 1:10. Subsequent analysis of the data was performed using a series of standard curves constructed with a range of standards, including bovine serum albumin (67 kDa), peroxidase (40.2 kDa), myoglobin (17 kDa), cytochrome C (12.4 kDa), and aprotinin (6.5 kDa).

### 3.5. Fourier Transform Infrared Spectroscopy (FTIR) of the PTP

The FTIR spectroscopy technique was employed in accordance with the methodology described by Wen et al. [34], with certain modifications being made. Lyophilized the PTP samples (5.0 mg) under different pH treatments (pH of 3.0, 5.0, 7.0, 9.0, 11.0), and with potassium bromide (KBr) (100 mg) were pressed into thin slices. FTIR spectra were acquired for the processed thin sections utilizing an infrared spectroscopy instrument (Nicolet iS10, Thermo Fisher Scientific, Waltham, MA, USA), with a 32 scan acquisition per sample, at a resolution of 4 cm^−1^ over a scanning range of 4000 to 400 cm^−1^ at 25 °C. The secondary structure composition of the PTP was calculated based on FTIR spectra using a linear secondary fitting with Peakfit V4.12.

### 3.6. Intrinsic Fluorescence Spectroscopy

Endogenous fluorescence spectra were obtained via a fluorescence spectrometer (F2700, Hitachi, Tokyo, Japan). The method employed was based on that of Jian et al. [27] but with appropriate adjustments. The PTP dispersion was pH-adjusted to 3.0, 5.0, 7.0, 9.0, and 11.0 while maintaining a concentration of 30 µg/mL. The protein dispersions were stirred for 1 h using a magnetic stirrer at room temperature. Aliquots (3 mL) of the PTP at different pH levels were scanned in the range of 300–540 nm. The excitation wavelength and slit width were set to 280 nm and 10 nm, respectively.

### 3.7. Surface Hydrophobicity

As previously mentioned by Lv et al. [35], the pH of the PTP dispersion at a concentration of 30 mg/mL was adjusted to 2–13 and then magnetically stirred for one hour at room temperature. Briefly, 4 mL of this dispersion was mixed with 20 µL of ANS (1-anilino-8-naphthalene sulfonic acid). The obtained mixtures were examined by using a fluorescence spectrophotometer (Hitachi F2700; Tokyo, Japan) to assess surface hydrophobicity. The gained mixtures were vortexed evenly for 5 s and scanned in the range of 394–728 nm using a fluorescence spectrophotometer (F2700, Hitachi, Tokyo, Japan). The slit width was 5 nm, and the excitation and emission wavelengths were 394 and 728 nm, respectively.

### 3.8. Free Sulfhydryl Groups (SH) of the PTP

The SH of the PTP at different pH levels was determined according to the method described by Wenxin et al. [36]. The pH of 20 mg/mL of the PTP was first adjusted to 2.0–13.0 and magnetically stirred for 1 h. Then, 2 mL of the PTP dispersion was mixed with 2 mL of tri-glycine buffer, and the mixture was quickly mixed with 50 μL of Ellman’s reagent. Subsequently, the mixture was placed in the dark at 25 degrees Celsius for 20 min and analyzed using a UV–visible spectrophotometer (UV-1900, Shanghai eSpectrum Instruments Co., Ltd., Shanghai, China) at 412 nm to determine the absorbance of the solution. The free sulfhydryl content was then calculated utilizing Equation (1).

(1) $$\begin{array}{c} SH\left(µmol/L\right)=\frac{73.53\times A_{412}\times D}{C} \end{array}$$

where A_412_ is the absorbance at 412 nm, C is the concentration of the sample (mg/mL), D is the coefficient of dilution (10), 73.53 is derived from 10^6^/1.36 × 10^−4^, and 1.36 × 10^−4^ is the molar absorbance of Ellman’s reagent.

### 3.9. Particle Size Distribution and Zeta Potential

The pH of the PTP dispersions (5 mg/mL) was adjusted to pH values of 3.0, 5.0, 7.0, 9.0, and 11.0, and the protein dispersions were stirred for 1 h using a magnetic stirrer at room temperature. The particle size distribution and zeta potential of the PTP dispersions were determined using a laser particle size analyzer (Zetasizer Nano ZS90, Malvern Instruments Ltd., Malvern, UK).

### 3.10. Solubility

The PTP solubilities were determined by a slightly modified method described by Yigang et al. [37]. After adjusting the pH to the range of 2.0–13.0, the PTP was dissolved in deionized water (10:1, W/V) and agitated for 30 min prior to centrifugation (6000 rpm, 15 min). Employing the Bradford method [38], the concentration of protein in both the initial protein solution and the supernatant was ascertained. Equation (2) was used to calculate the PTP’s solubility.

(2) $$\begin{array}{c} Solubility(\%)=\frac{P_{t}}{P_{s}}\times 100 \end{array}$$

where P_s_ is the PTP concentration determined by the Thomas Brilliant Blue method and P_t_ is the total protein of the sample. 

### 3.11. Foaming Properties

The foaming capacity (FC) and foaming stability (FS) of the PTP were measured after 10 mg/mL of the PTP’s pH was brought to 2.0–13.0 and stirred magnetically for 1 h at room temperature, according to the method described by Farjana [39]. Homogenization (10,000 r, 90 s) was performed using a homogenizer (T25, IKA, Staufen, Germany). The initial foam volume as well as the foam volumes were recorded at 0 and 30 min. The PTP’s FC and FS were determined using Equations (3) and (4).

(3) $$\begin{array}{c} FC\left(\%\right)=\frac{V_{0}}{V}\times 100 \end{array}$$

(4) $$\begin{array}{c} FS\left(\%\right)=\frac{V_{1}}{V_{0}}\times 100 \end{array}$$

where V_0_ (mL) and V_1_ (mL) are the volumes of the foam at 0 min and 30 min, respectively, and V (mL) is the initial volume.

### 3.12. Emulsification Properties

The pH of 1 mg/mL of the PTP was adjusted to 2.0–13.0 and stirred magnetically for 1 h at room temperature. The emulsification properties were evaluated according to the method described by Fan et al. [40]. Soybean oil and the PTP emulsion were mixed in a ratio of 1:3 (W/W) using a homogenizer (10,000 r, 90 s). Immediately after mixing, 10 μL was taken from the bottom of the emulsion and mixed with 10 mL of 0.1% SDS. The absorbance of the diluted emulsion was quantified at 500 nm using a UV–visible spectrophotometer, and the above steps were repeated after 10 min. The EAI and ESI of the PTP were calculated using Equations (5) and (6), respectively.

(5) $$\begin{array}{c} ESI\left(min\right)=\frac{A_{0}}{A_{0}-A_{10}}\times 10 \end{array}$$

(6) $$\begin{array}{c} EAI\left(m^{2}/g\right)=\frac{DF\times 2\times 2.303\times A_{0}}{C\times ɸ\times 10000} \end{array}$$

A_0_ and A_10_ are the extinction of the diluted emulsion at 0 and 10 min, respectively, DF is the density of dilution (1000), c is the concentration of protein (g/mL), φ is the volume fraction of oil (0.25), and t is the time (10 min). 

### 3.13. Thermal Properties of the PTP

According to the method described by Zhu et al. [41], the thermal properties of the PTP were evaluated. Differential calorimetric scanning was carried out by placing 2 mg of the lyophilized PTP samples under different pH treatments (pH of 3.0, 5.0, 7.0, 9.0, 11.0) in an aluminum dish and heating it at a rate of 5 °C per minute between 30 °C and 200 °C (TA Instruments Thermal Analysis, New Castle, DE, USA).

### 3.14. Antioxidant Properties of the PTP

#### 3.14.1. ABTS Radical Scavenging Activity

The ABTS solution was prepared by mixing 7 mM ABTS and 2.45 mM potassium persulfate solution in a ratio of 1:0.5. The mixture was then stored in the dark for 12–16 h at room temperature. The solution was diluted with phosphate buffer (pH of 7.4) to an optical density of 0.700 ± 0.02 cm^−1^ at 734 nm. The lyophilized PTP powder was prepared as dispersions at concentrations of 0.1, 0.2, 0.3, 0.4, 0.5, 0.6, 0.7, 0.8, 0.9, and 1.0 mg/mL using phosphate buffer solution. Then, 1 mL of the different concentrations of the dispersion was mixed with 4 mL of 1 mL ABTS solution, followed by incubation for 6 min at room temperature. The ABTS radical scavenging activity of the PTP was calculated using Equation (7).

(7) $$\begin{array}{c} ABTS\;radicai\;scavening\;activity\;\left(\%\right)=\frac{A_{0}-A_{1}}{A_{0}}\times 100 \end{array}$$

where A_1_ is the sample with the ABTS solution and A_0_ is deionized water with the ABTS solution.

#### 3.14.2. DPPH Radical Scavenging Activity

The lyophilized PTP powder was prepared as dispersions at concentrations of 0.1, 0.2, 0.3, 0.4, 0.5, 0.6, 0.7, 0.8, 0.9, and 1.0 mg/mL using phosphate buffer solution. The sample was incubated for 30 min at 25 °C in the dark with an equal volume of 0.1 mmol/L DPPH solution (prepared with 95% ethanol). The absorbance was then measured at 517 nm (As). The DPPH radical scavenging activity of the PTP was calculated using the following Equation (8).

(8) $$\begin{array}{c} DPPH\;radical\;scavenging\;activity\;\left(\%\right)=\frac{A_{1}-A_{S}+A_{0}}{A_{1}}\times 100 \end{array}$$

where A_0_ and A_1_ are the absorbencies of 95% ethanol used to substitute the sample dispersion and 95% ethanol used to replace DPPH, respectively.

### 3.15. Statistical Analysis

All the studies were performed in triplicate, and the average and the mean standard error were calculated. Statistical analysis was performed using Origin 2021 software, and IBM SPSS Statistics 26 was used to determine whether significant differences existed. A *p*-value < 0.05 was considered statistically significant.

## 4. Conclusions

A strong positive correlation was observed between the alkaline pH shift and the solubility of the PTP. This correlation was attributed to the increased thermal stability and hydrophobic interactions of the alkali-treated PTP. The particle size of the acidic pH-shifted PTP samples was larger, and the absolute value of zeta potential was smaller, resulting in reduced foaming and emulsification. In contrast, alkaline pH shift treatments resulted in smaller particle sizes, larger zeta potentials, enhanced foaming and emulsification, and exposure of more free sulfhydryl groups. The secondary structure of the PTP underwent a transition from an irregular, disordered state to an ordered state, as evidenced by a reduction in the number of irregular curls. Similarly, the tertiary structure of the PTP underwent a transformation, including a notable reduction in fluorescence intensity. The present study demonstrated that pH adjustment could effectively alter the physicochemical properties, solubility, foaming ability, and emulsification of delta-brown fingerling protein, among other properties.

## Figures and Tables

**Table 1:** Amino acid composition of the PTP.

Amino Acid	(g/100 g)	FAO/WHO Recommendation for Children (g/100 g)	FAO/WHO Recommendation for Adults (g/100 g)
Threonine (Thr)	4.83 ± 0.21	3.10	2.50
Valine (Val)	5.39 ± 0.17	4.30	4.00
Cysteine (Cys)	2.92 ± 0.30		
Methionine (Met)	3.29 ± 0.20		
Isoleucine (Ile)	4.13 ± 0.04	3.20	3.00
Leucine (Leu)	6.62 ± 0.08	6.60	6.10
Tyrosine (Tyr)	4.58 ± 0.13		
Phenylalanine (Phe)	5.66 ± 0.09		
Lysine (Lys)	4.87 ± 0.11	5.70	4.80
Histidine (His)	3.12 ± 0.06	2.00	1.60
Asparagine (Asp)	8.09 ± 0.31	-	-
Serine (Ser)	4.98 ± 0.20	-	-
Glutarnine (Glu)	8.26 ± 0.16	-	-
Glycine (Gly)	4.57 ± 0.24	-	-
Alaine (Ala)	5.28 ± 0.14	-	-
Arginine (Arg)	4.65 ± 0.18	-	-
Proline (Pro)	4.31 ± 0.07	-	-
Essential amino acid	45.44		
Nonessential amino acid	40.15		
Hydrophobic	17.33		
Hydrophilic	39.25		
Acidic	16.36		
Basic	12.65		
Aromatic	10.24	5.2	4.1
Branched-chain	16.15		
Negatively charged	12.65		
Positively charged	26.17		
SAA(Cys+Met)	6.21	2.7	2.3

**Table 2:** Thermal properties of the PTP at pH 3, 5, 7, 9, and 11.

	T_O_ (°C)	T_P_ (°C)	ΔH (J/g)
pH3	43.54 ± 0.32 ^a^	68.44 ± 0.12 ^d^	42.48 ± 0.47 ^d^
pH5	40.57 ± 2.04 ^b^	71.19 ± 0.67 ^c^	90.52 ± 0.09 ^c^
pH7	42.60 ± 1.79 ^ab^	71.07 ± 0.17 ^c^	35.17 ± 0.70 ^e^
pH9	41.51 ± 1.79 ^a^	74.36 ± 0.93 ^b^	137.59 ± 0.54 ^a^
pH11	43.82 ± 0.85 ^a^	77.66 ± 0.14 ^a^	93.34 ± 0.31 ^b^

Different letters represent significant (*p* ≤ 0.05) differences between groups.

**Table 3:** Zeta potential and average particle size of the PTP at pH 3, 5, 7, 9, and 11.

	Average Particle Size (nm)	Zeta Potential (mV)
pH3	3199.84 ± 0.47 ^e^	−6.03 ± 0.47 ^a^
pH5	1841.70 ± 0.73 ^d^	−26.78 ± 0.22 ^b^
pH7	1463.05 ± 0.46 ^c^	−34.23 ± 0.67 ^c^
pH9	1245.21 ± 0.91 ^b^	−36.60 ± 0.86 ^d^
pH11	1115.11 ± 0.57 ^a^	−45.33 ± 0.54 ^e^

Different letters represent significant (*p* ≤ 0.05) differences between groups.

## Data Availability

The datasets generated for this study are available upon request to the corresponding author.
